# The genome sequence of the thick-headed fly, *Myopa tessellatipennis* (Motschulsky, 1859)

**DOI:** 10.12688/wellcomeopenres.19108.1

**Published:** 2023-03-10

**Authors:** Michael Ashworth, Steven Falk, Natural History Museum Genome Acquisition Lab, University of Oxford and Wytham Woods Genome Acquisition Lab, Darwin Tree of Life Barcoding collective, Wellcome Sanger Institute Tree of Life programme, Wellcome Sanger Institute Scientific Operations: DNA Pipelines collective, Tree of Life Core Informatics collective, David K. Clements, Darwin Tree of Life Consortium

**Affiliations:** 1 Independent Researcher, Yeovil, Somerset, UK; 2 Independent Researcher, Kenilworth, Warwickshire, UK; 3 Conopid Recording Scheme for Britain & Ireland, Cardiff, Wales, UK

**Keywords:** Myopa tessellatipennis, thick-headed fly, genome sequence, chromosomal, Diptera, Conopidae

## Abstract

We present a genome assembly from an individual female *Myopa tessellatipennis* (Arthropoda; Insecta; Diptera; Conopidae). The genome sequence is 249.3 megabases in span. Most of the assembly is scaffolded into four chromosomal pseudomolecules, including the assembled X sex chromosome. The mitochondrial genome has also been assembled and is 18.3 kilobases in length.

## Species taxonomy

Eukaryota; Metazoa; Ecdysozoa; Arthropoda; Hexapoda; Insecta; Pterygota; Neoptera; Endopterygota; Diptera; Brachycera; Muscomorpha; Conopoidea; Conopidae; Myopinae; *Myopa*; *Myopa tessellatipennis* (Motschulsky, 1859) (NCBI:txid2829445).

## Background

*Myopa tessellatipennis* Motschulsky, 1859 (Diptera: Conopidae, Myopinae) is a member of the family commonly referred to as ‘thick-headed flies’, or more recently as ‘bee-grabbers’. As far as is known, all members of this family develop as endoparasitoids of other insects, although life history data for many species is lacking. Hosts primarily comprise species of aculeate Hymenoptera, except in the large and mainly tropical subfamily Stylogasterinae ( Stuke, 2017). Just over 800 valid species are currently recognised, occurring in all parts of the world except for the polar regions.

The genus *Myopa* currently comprises about 44 species, just over half of which are Palaearctic in distribution ( Stuke, 2017). *Myopa tessellatipennis* lies within the ‘polystigma-group’ which comprises about 10 species in the Palaearctic. Nine *Myopa* species are known to occur in Britain ( Smith, 1969), with *tessellatipennis* being confused under *polystigma* Rondani, 1857 until clarification ( Smith, 1970). The British species can be identified using the keys of ( Clements, 1995) or ( Smit, 2013). *Myopa tessellatipennis* is mainly confined to the southern half of Britain, occurring most commonly in the south and east of England (UK Conopid Recording Scheme database). Elsewhere it occurs throughout the Western Palaearctic.

Host data is lacking for most *Myopa* species but where known comprises bees of the genus *Andrena* (Hymenoptera: Andrenidae). Smit *et al.*, 2018 reported a record of *Myopa pellucida* Robineau-Desvoidy, 1830 identified by DNA barcoding from a larva found within *Andrena nitida* Müller, 1776, but most of the host-associations in the literature are tentative ( Stuke, 2017). *Myopa tessellatipennis* occurs in a wide variety of mainly lowland habitats and is thought to parasitise *Andrena barbilabris* Kirby, 1802 and *A. flavipes* Panzer, 1799 although other host species may also be involved ( Edwards, 1985; Nieuwenhuijsen, 2009; Stuke, 2017).

Typically, female *Myopa* lie in wait for a host bee at nectaring sites, grabbing it in flight. Wrapping its body around the bee, it uses a specialised ovipositor to prise open a gap between the bee’s sclerites to inject an egg in the abdominal cavity. The fly larva develops within the host and pupation occurs upon its death. Infestation by conopid larvae has been shown to significantly alter the foraging and other behaviour of the host (see Clements, 1997), in some cases by inducing fossorial behaviour in which the host digs into the ground to provide a protected overwintering site for the conopid pupa ( Malfi *et al.*, 2014; Müller, 1994). Most *Myopa* species fly in the spring or early summer, coinciding with their likely hosts, although a few also fly later in the season. The barcoding of conopids is desirable in tackling their complex taxonomy, and also in allowing the identification of larvae when encountered within their aculeate hosts, some of which are commercially important, for example as pollinators or in honey production.

**Figure 1.**
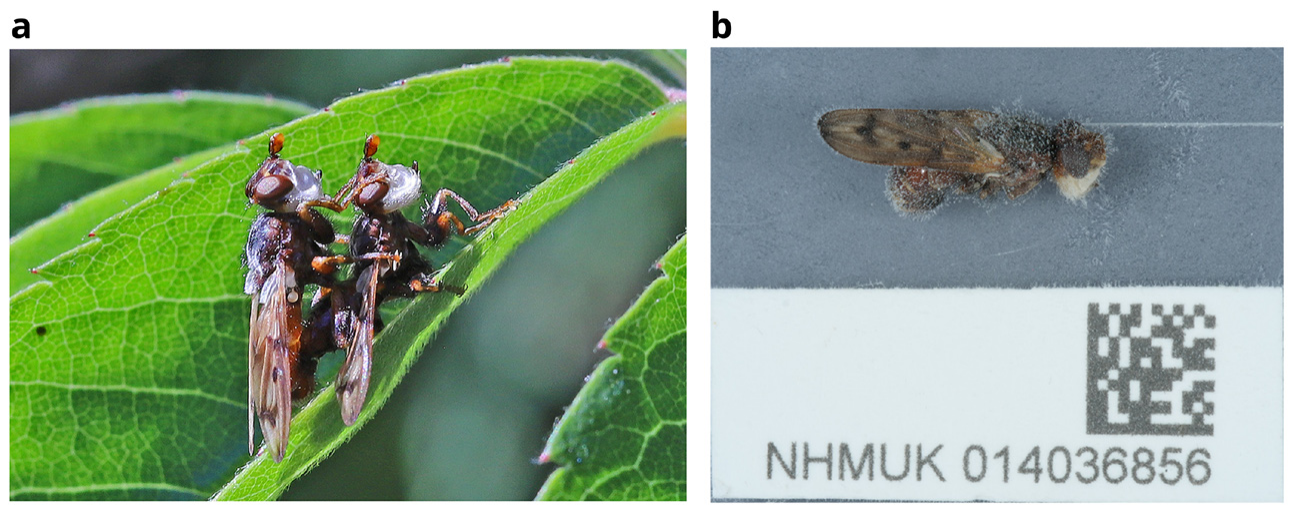
**a**) The mating pair of Myopa tessellatipennis, which were taken for genome analysis. The female of this pair (NHMUK014036856) was used for genome assembly. **b**) Photograph of the *Myopa tessellatipennis* (idMyoTess1) specimen used for genome sequencing during preservation and processing.

A mating pair of *Myopa tessellatipennis* ( Figure 1) was observed on 26 April 2021 in a rural garden in Somerset, south-west England, and the pair were sent live to the Natural History Museum, London. The high-quality genome sequence for a female *M. tessellatipennis* reported here has been generated as part of the Darwin Tree of Life project. It will aid in understanding the biology, physiology and ecology of the species.

## Genome sequence report

The genome was sequenced from one female *Myopa tessellatipennis* ( Figure 1) collected from Yeovil, Somerset, England, UK (latitude 50.97, longitude –2.68). A total of 77-fold coverage in Pacific Biosciences single-molecule HiFi long reads was generated. Primary assembly contigs were scaffolded with chromosome conformation Hi-C data. Manual assembly curation corrected six missing or mis-joins reducing the scaffold number by 54.55%.

**Figure 2.**
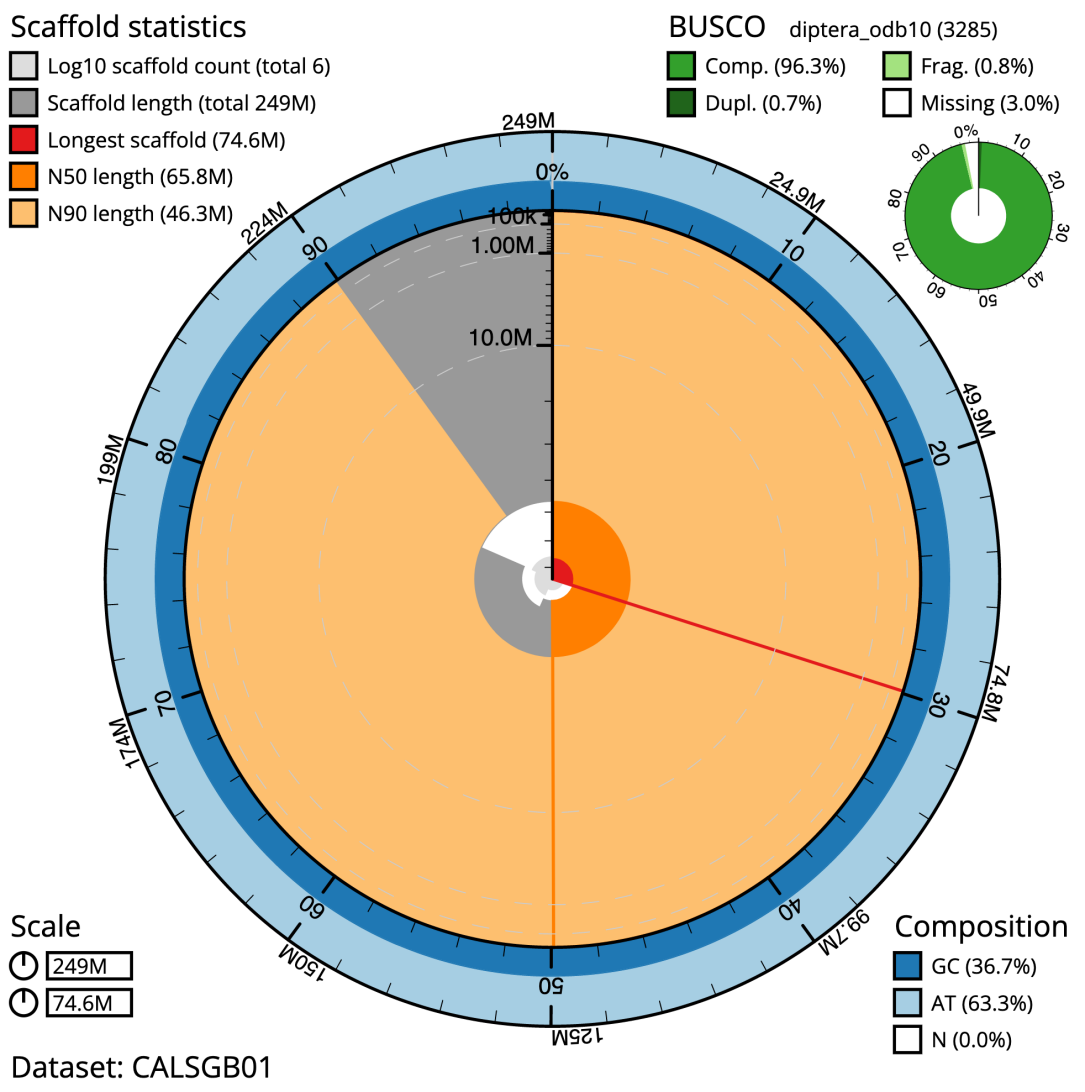
Genome assembly of *Myopa tessellatipennis*, idMyoTess1.1: metrics. The BlobToolKit Snailplot shows N50 metrics and BUSCO gene completeness. The main plot is divided into 1,000 size-ordered bins around the circumference with each bin representing 0.1% of the 249,275,748 bp assembly. The distribution of scaffold lengths is shown in dark grey with the plot radius scaled to the longest scaffold present in the assembly (74,632,285 bp, shown in red). Orange and pale-orange arcs show the N50 and N90 scaffold lengths (65,812,558 and 46,318,963 bp), respectively. The pale grey spiral shows the cumulative scaffold count on a log scale with white scale lines showing successive orders of magnitude. The blue and pale-blue area around the outside of the plot shows the distribution of GC, AT and N percentages in the same bins as the inner plot. A summary of complete, fragmented, duplicated and missing BUSCO genes in the diptera_odb10 set is shown in the top right. An interactive version of this figure is available at https://blobtoolkit.genomehubs.org/view/idMyoTess1.1/dataset/CALSGB01/snail.

The final assembly has a total length of 249.3 Mb in five sequence scaffolds with a scaffold N50 of 65.8 Mb ( Table 1). Most (99.99%) of the assembly sequence was assigned to four chromosomal-level scaffolds, representing three autosomes, and the X sex chromosome. Chromosome-scale scaffolds confirmed by the Hi-C data are named in order of size ( Figure 2– Figure 5; Table 2). The assembly has a BUSCO v5.3.2 ( Manni *et al.*, 2021) completeness of 96.3% (single 95.6%, duplicated 0.7%) using the diptera_odb10 reference set. While not fully phased, the assembly deposited is of one haplotype. Contigs corresponding to the second haplotype have also been deposited.

**Table 1.** Genome data for *Myopa tessellatipennis*, idMyoTess1.1.

Project accession data		
Assembly identifier	idMyoTess1.1	
Species	*Myopa tessellatipennis*	
Specimen	idMyoTess1	
NCBI taxonomy ID	2829445	
BioProject	PRJEB52797	
BioSample ID	SAMEA9654265	
Isolate information	idMyoTess1, female: abdomen (PacBio sequencing), head (Hi-C) idMyoTess2, male: whole organism (RNA-Seq)	
Assembly metrics *		*Benchmark*
Consensus quality (QV)	68	*≥ 50*
*k*-mer completeness	100%	*≥ 95%*
BUSCO **	C:96.3%[S:95.6%,D:0.7%], F:0.8%,M:3.0%,n:3,285	*C ≥ 95%*
Percentage of assembly mapped to chromosomes	99.99%	*≥ 95%*
Sex chromosomes	X chromosome	*localised homologous pairs*
Organelles	Mitochondrial genome assembled	*complete single alleles*
Raw data accessions		
PacificBiosciences SEQUEL II	ERR9793195	
Hi-C Illumina	ERR9730867	
PolyA RNA-Seq Illumina	ERR10123700	
Genome assembly		
Assembly accession	GCA_943737955.1	
*Accession of alternate haplotype*	GCA_943737945.1	
Span (Mb)	249.3	
Number of contigs	18	
Contig N50 length (Mb)	39.9	
Number of scaffolds	5	
Scaffold N50 length (Mb)	65.8	
Longest scaffold (Mb)	74.6	

* Assembly metric benchmarks are adapted from column VGP-2020 of “Table 1: Proposed standards and metrics for defining genome assembly quality” from ( Rhie *et al.*, 2021). ** BUSCO scores based on the diptera_odb10 BUSCO set using v5.3.2. C = complete [S = single copy, D = duplicated], F = fragmented, M = missing, n = number of orthologues in comparison. A full set of BUSCO scores is available at https://blobtoolkit.genomehubs.org/view/idMyoTess1.1/dataset/CALSGB01/busco.

**Figure 3.**
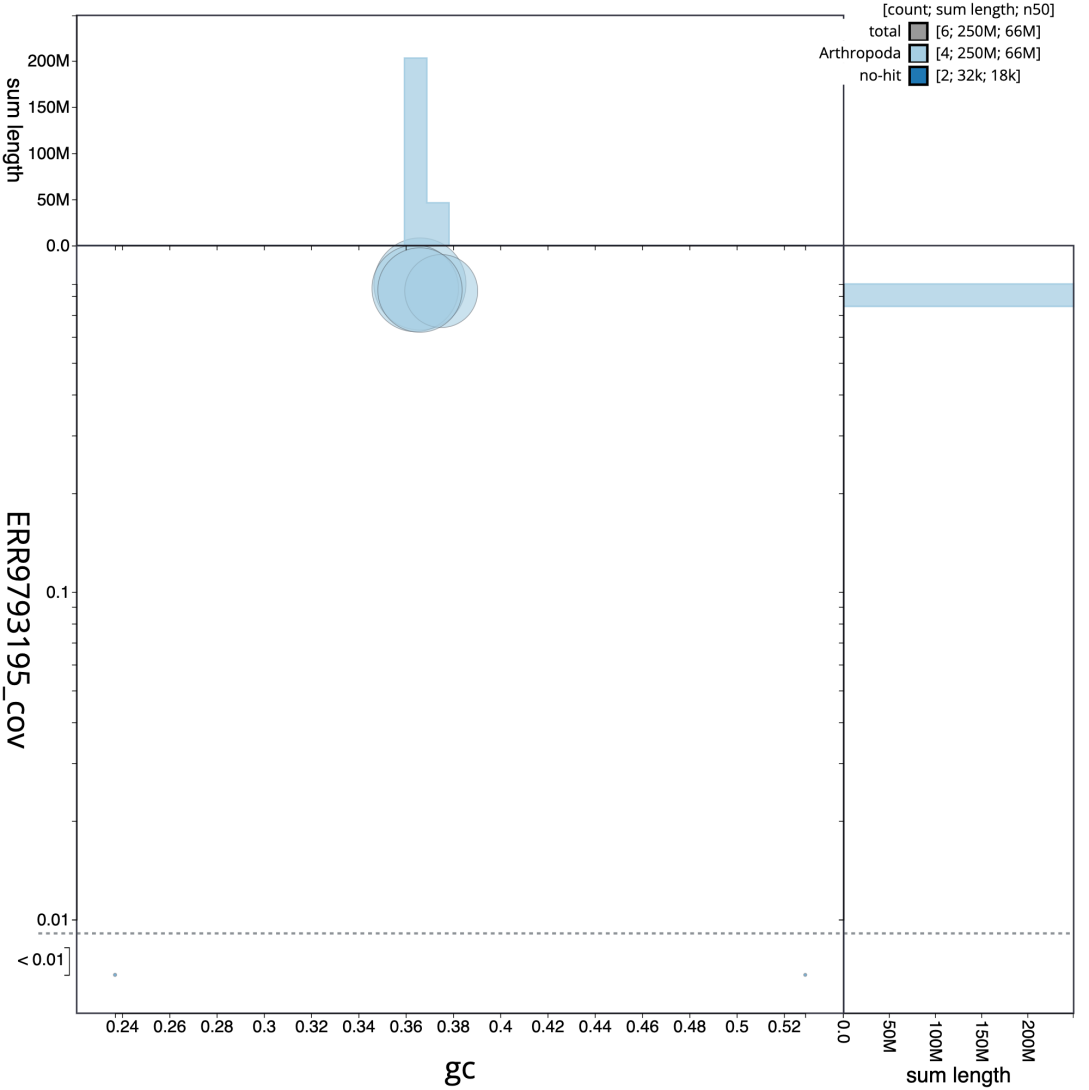
Genome assembly of *Myopa tessellatipennis*, idMyoTess1.1: GC coverage. BlobToolKit GC-coverage plot. Scaffolds are coloured by phylum. Circles are sized in proportion to scaffold length. Histograms show the distribution of scaffold length sum along each axis. An interactive version of this figure is available at https://blobtoolkit.genomehubs.org/view/idMyoTess1.1/dataset/CALSGB01/blob.

**Figure 4.**
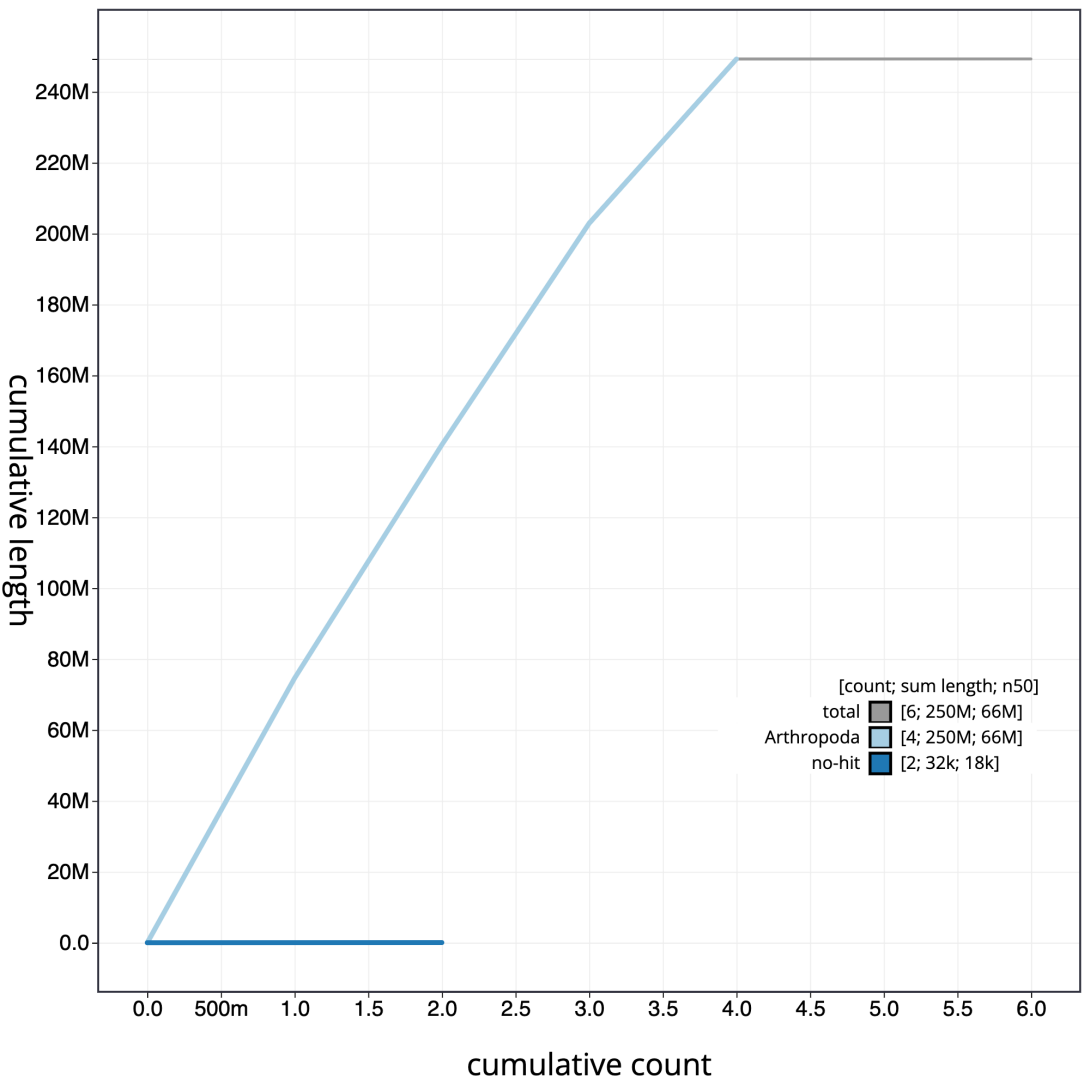
Genome assembly of *Myopa tessellatipennis*, idMyoTess1.1: cumulative sequence. BlobToolKit cumulative sequence plot. The grey line shows cumulative length for all scaffolds. Coloured lines show cumulative lengths of scaffolds assigned to each phylum using the buscogenes taxrule. An interactive version of this figure is available at https://blobtoolkit.genomehubs.org/view/idMyoTess1.1/dataset/CALSGB01/cumulative.

**Figure 5.**
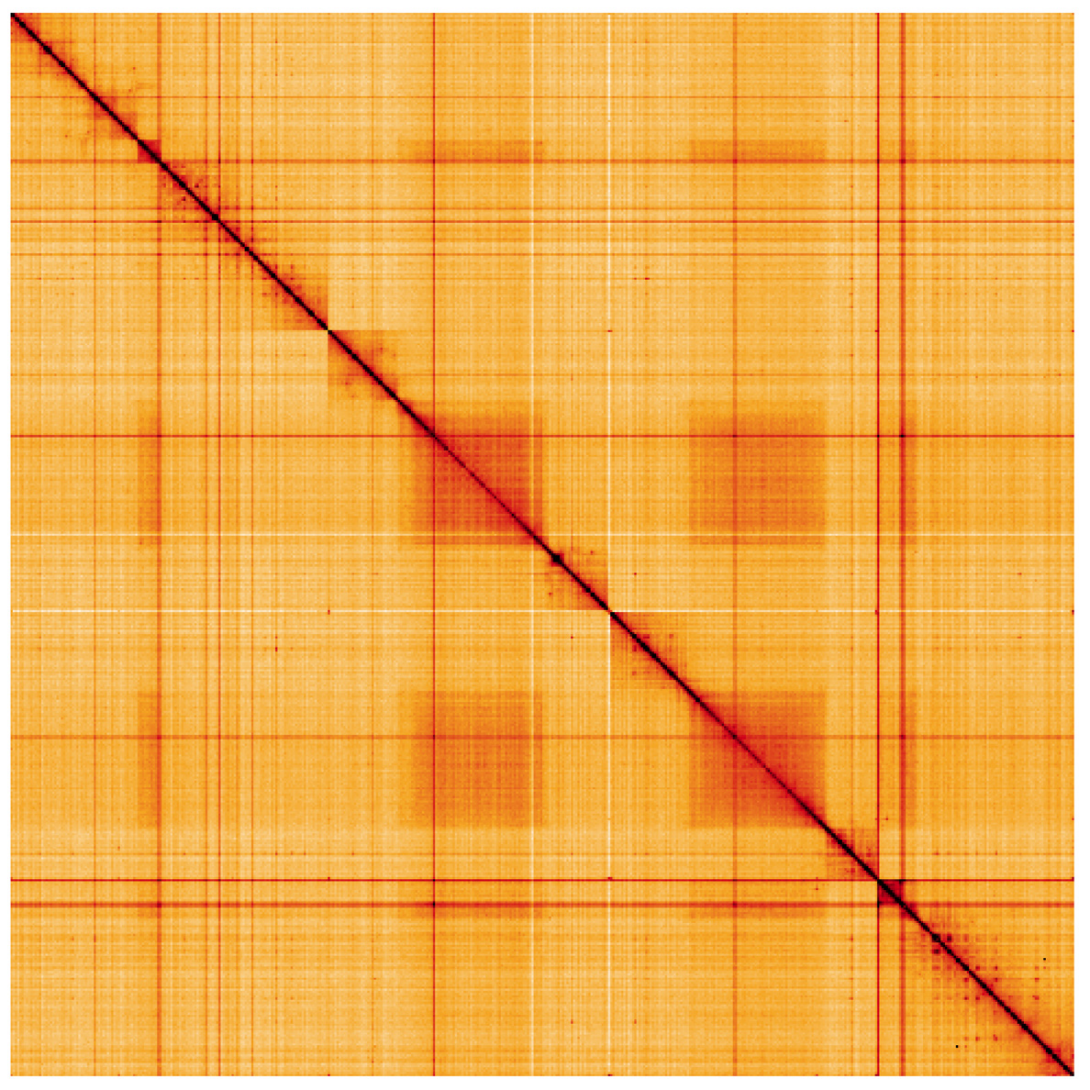
Genome assembly of *Myopa tessellatipennis*, idMyoTess1.1: Hi-C contact map. Hi-C contact map of the idMyoTess1.1 assembly, visualised using HiGlass. Chromosomes are shown in order of size from left to right and top to bottom. An interactive version of this figure may be viewed at https://genome-note-higlass.tol.sanger.ac.uk/l/?d=dDPd6svVRwScfXFIRd8dMg.

**Table 2.** Chromosomal pseudomolecules in the genome assembly of *Myopa tessellatipennis*, idMyoTess1.

INSDC accession	Chromosome	Size (Mb)	GC%
OX031314.1	1	74.63	36.6
OX031315.1	2	65.81	36.4
OX031317.1	3	46.32	37.5
OX031316.1	X	62.48	36.6
OX031318.1	MT	0.02	23.7

## Methods

### Sample acquisition and nucleic acid extraction

A female *Myopa tessellatipennis* (idMyoTess1) was collected from Yeovil, Somerset, England, UK (latitude 50.97, longitude –2.68) on 26 April 2021. The specimen was taken from a rural garden by Mike Ashworth (independent researcher) using an aerial net. The specimen was identified by Mike Ashworth and preserved at –80°C. This specimen was used for PacBio and Hi-C analysis.

A male specimen (idMyoTess2) was collected from Wytham Farm, Oxfordshire (latitude 51.79, longitude –1.32) on 19 April 2021 by netting. The specimen was collected and identified by Steven Falk (independent researcher), and was preserved on dry ice. This specimen was used for RNA sequencing.

DNA was extracted at the Tree of Life laboratory, Wellcome Sanger Institute (WSI). The idMyoTess1 sample was weighed and dissected on dry ice with tissue set aside for Hi-C sequencing. Abdomen tissue was cryogenically disrupted to a fine powder using a Covaris cryoPREP Automated Dry Pulveriser, receiving multiple impacts. High molecular weight (HMW) DNA was extracted using the Qiagen MagAttract HMW DNA extraction kit. HMW DNA was sheared into an average fragment size of 12–20 kb in a Megaruptor 3 system with speed setting 30. Sheared DNA was purified by solid-phase reversible immobilisation using AMPure PB beads with a 1.8X ratio of beads to sample to remove the shorter fragments and concentrate the DNA sample. The concentration of the sheared and purified DNA was assessed using a Nanodrop spectrophotometer and Qubit Fluorometer and Qubit dsDNA High Sensitivity Assay kit. Fragment size distribution was evaluated by running the sample on the FemtoPulse system.

RNA was extracted from idMyoTess2 in the Tree of Life Laboratory at the WSI using TRIzol, according to the manufacturer’s instructions. RNA was then eluted in 50 μl RNAse-free water and its concentration assessed using a Nanodrop spectrophotometer and Qubit Fluorometer using the Qubit RNA Broad-Range (BR) Assay kit. Analysis of the integrity of the RNA was done using Agilent RNA 6000 Pico Kit and Eukaryotic Total RNA assay.

### Sequencing

Pacific Biosciences HiFi circular consensus and 10X Genomics read cloud DNA sequencing libraries were constructed according to the manufacturers’ instructions. Poly(A) RNA-Seq libraries were constructed using the NEB Ultra II RNA Library Prep kit. DNA and RNA sequencing was performed by the Scientific Operations core at the WSI on Pacific Biosciences SEQUEL II (HiFi) and Illumina NovaSeq 6000 (RNA-Seq) instruments. Hi-C data were also generated from head tissue of idMyoTess1 using the Arima v2 kit and sequenced on the Illumina NovaSeq 6000 instrument.

### Genome assembly

Assembly was carried out with Hifiasm ( Cheng *et al.*, 2021) and haplotypic duplication was identified and removed with purge_dups ( Guan *et al.*, 2020). The assembly was then scaffolded with Hi-C data ( Rao *et al.*, 2014) using YaHS ( Zhou *et al.*, 2023). The assembly was checked for contamination as described previously ( Howe *et al.*, 2021). Manual curation was performed using HiGlass ( Kerpedjiev *et al.*, 2018) and Pretext ( Harry, 2022). The mitochondrial genome was assembled using MitoHiFi ( Uliano-Silva *et al.*, 2022), which performed annotation using MitoFinder ( Allio *et al.*, 2020). The genome was analysed and BUSCO scores generated within the BlobToolKit environment ( Challis *et al.*, 2020). Table 3 contains a list of all software tool versions used, where appropriate.

**Table 3.** Software tools and versions used.

Software tool	Version	Source
BlobToolKit	3.4.0	Challis *et al.*, 2020
Hifiasm	0.16.1-r375	Cheng *et al.*, 2021
HiGlass	1.11.6	Kerpedjiev *et al.*, 2018
MitoHiFi	2	Uliano-Silva *et al.*, 2022
PretextView	0.2	Harry, 2022
purge_dups	1.2.3	Guan *et al.*, 2020
YaHS	yahs-1.1.91eebc2	Zhou *et al.*, 2023

### Ethics and compliance issues

The materials that have contributed to this genome note have been supplied by a Darwin Tree of Life Partner. The submission of materials by a Darwin Tree of Life Partner is subject to the Darwin Tree of Life Project Sampling Code of Practice. By agreeing with and signing up to the Sampling Code of Practice, the Darwin Tree of Life Partner agrees they will meet the legal and ethical requirements and standards set out within this document in respect of all samples acquired for, and supplied to, the Darwin Tree of Life Project. All efforts are undertaken to minimise the suffering of animals used for sequencing. Each transfer of samples is further undertaken according to a Research Collaboration Agreement or Material Transfer Agreement entered into by the Darwin Tree of Life Partner, Genome Research Limited (operating as the Wellcome Sanger Institute), and in some circumstances other Darwin Tree of Life collaborators.

## Data Availability

European Nucleotide Archive: *Myopa tessellatipennis*. Accession number PRJEB52797; https://identifiers.org/ena.embl/PRJEB52797. ( Wellcome Sanger Institute, 2022) The genome sequence is released openly for reuse. The *Myopa tessellatipennis* genome sequencing initiative is part of the Darwin Tree of Life (DToL) project. All raw sequence data and the assembly have been deposited in INSDC databases. The genome will be annotated using available RNA-Seq data and presented through the Ensembl pipeline at the European Bioinformatics Institute. Raw data and assembly accession identifiers are reported in Table 1.

## Author information

Members of the Natural History Museum Genome Acquisition Lab are listed here: https://doi.org/10.5281/zenodo.4790043.

Members of the University of Oxford and Wytham Woods Genome Acquisition Lab are listed here: https://doi.org/10.5281/zenodo.4789928.

Members of the Darwin Tree of Life Barcoding collective are listed here: https://doi.org/10.5281/zenodo.4893703.

Members of the Wellcome Sanger Institute Tree of Life programme are listed here: https://doi.org/10.5281/zenodo.4783585.

Members of Wellcome Sanger Institute Scientific Operations: DNA Pipelines collective are listed here: https://doi.org/10.5281/zenodo.4790455.

Members of the Tree of Life Core Informatics collective are listed here: https://doi.org/10.5281/zenodo.5013541.

Members of the Darwin Tree of Life Consortium are listed here: https://doi.org/10.5281/zenodo.4783558.
