# Supplementary material for: The genome sequence of the thick-headed fly,  Myopa tessellatipennis (Motschulsky, 1859)
Source: Wellcome Open Res. 2023 Mar 10;8:115. [Version 1] doi: 10.12688/wellcomeopenres.19108.1 (PMC10468684; doi:10.12688/wellcomeopenres.19108.1)
Supplement: Peer review history

# Peer review: Reviewer response for version 1

**DOI:** 10.21956/wellcomeopenres.21184.r65679

**Published:** 2023-08-30

**Authors:** Stephen Richards

**Affiliations:** 1 Human Genome Sequencing Center, Baylor College of Medicine, Houston, TX, USA

What fun! - this is a fascinating understudied parasitic family,  and I hope that this high quality reference sequence will provide one new pathway to learning more about these species.

 As always the Darwin tree of life project has generated an excellent genome reference that will stand the test of time, and with the other genomes be a foundation of biological research in the 21st century and beyond. I am also impressed and pleased that the DToL is collaborating with entomologists beyond the usual research networks given the importance of this project for all humanity in future years. 

 In terms of bioinformatic methods, they are well documented, but it would be nice if the assembly pipeline was made public on the various public commercial cloud services - a request for the larger project and beyond the scope of this publication.

 Another nice touch is that I had no issues finding the reference sequence in the NCBI and EBI public databases. Once again I thank the project for making all the data publicly available as rapidly as possible. 

 The authors and project as a whole are to be commended on generating a wonderful reference for the community.

Are sufficient details of methods and materials provided to allow replication by others?

Partly

Is the rationale for creating the dataset(s) clearly described?

Yes

Are the datasets clearly presented in a useable and accessible format?

Yes

Are the protocols appropriate and is the work technically sound?

Yes

Reviewer Expertise:

Drosophila Genetics, Human Genetics, Arthropod Genomics, Earth BioGenome Project

I confirm that I have read this submission and believe that I have an appropriate level of expertise to confirm that it is of an acceptable scientific standard.

# Peer review: Reviewer response for version 1

**DOI:** 10.21956/wellcomeopenres.21184.r59759

**Published:** 2023-07-10

**Authors:** Jan Ševčík

**Affiliations:** 1 University of Ostrava, Ostrava, Czech Republic

The first genomic data are presented for the relatively rare fly *Myopa tessellatipennis*. Both chromosome and mitogenome assemblies appear as of high quality. I have only following minor comment.

 Full taxonomic name of the species is *Myopa tessellatipennis* Motschulsky, 1859 as stated in the Background chapter but not *Myopa tessellatipennis* (Motschulsky, 1859) as is used in the title and elsewhere in the text. Please correct.

 It is not clear from the text if the entire body of the specimen was used for DNA or RNA isolation. This should be specified in detail. I strongly recommend to keep at least one wing and male or female terminalia as vouchers for further study and not to destroy the entire abdomen (or body). It is also useful to take (before DNA/RNA isolation) detailed photographs of all important morphological characters used for the taxonomic identification of the specimen(s) and include them in the paper. Habitus photo of mating specimens may be useful to attract attention of potential readers but not to substantiate exact identification of the species.

 Why a male from different locality was used for RNA isolation and not the male from the mating couple on the photograph? Please provide more details about that.

 Why RNA was isolated? Are transcriptomic data available? This should be commented on in the text.

Are sufficient details of methods and materials provided to allow replication by others?

Partly

Is the rationale for creating the dataset(s) clearly described?

Yes

Are the datasets clearly presented in a useable and accessible format?

Yes

Are the protocols appropriate and is the work technically sound?

Yes

Reviewer Expertise:

Diptera taxonomy; molecular phylogenetics; DNA barcoding

I confirm that I have read this submission and believe that I have an appropriate level of expertise to confirm that it is of an acceptable scientific standard.
